# Supplementary material for: Presenilin 1 Modulates Acetylcholinesterase Trafficking and Maturation
Source: Int J Mol Sci. 2023 Jan 11;24(2):1437. doi: 10.3390/ijms24021437 (PMC9864477; doi:10.3390/ijms24021437)
Supplement: Supplementary file 1 [file ijms-24-01437-s001.zip › Supplemental Fig v2/Supplemental Figure S1.pdf]

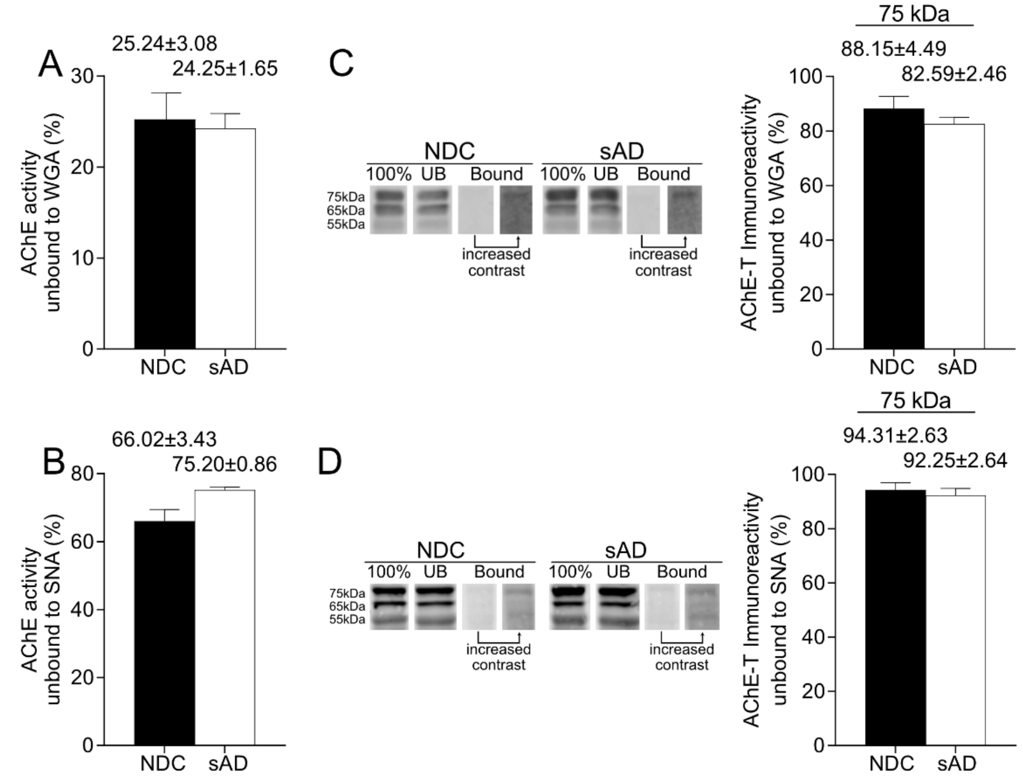


**Supplemental Figure S1. Lectin-binding analyses of brain cortices from Alzheimer's Disease (AD, n=4) patients compared to non-demented controls (NDC, n=4)**. Brain extracts were incubated with *Sambuccus nigra* (SNA) and *Wheat Germ Agglutinin* (WGA) lectins. Then unbound fraction was separated of bound fraction by centrifugation. The percentage over the total of AChE enzymatic activity not recognized by WGA (**A**) and SNA (**B**) was calculated. Binding of AChE protein was analysed by western blotting and the percentage of the 75-kDa immunoreactive AChE band that does not bind to the lectin respect to the AChE protein prior to lectin binding (100%) was calculated. Representative western blot for WGA (**C**) and SNA (**D**). An insert with more contrasted images of the bound fraction is included. To the right, densitometric quantification of bound immunoreactive 75-kDa band. The graphs represent mean ± SEM.
