# Supplementary material for: Presenilin 1 Modulates Acetylcholinesterase Trafficking and Maturation
Source: Int J Mol Sci. 2023 Jan 11;24(2):1437. doi: 10.3390/ijms24021437 (PMC9864477; doi:10.3390/ijms24021437)
Supplement: Supplementary file 1 [file ijms-24-01437-s001.zip › Supplemental Fig v2/Supplemental Figure S2.pdf]

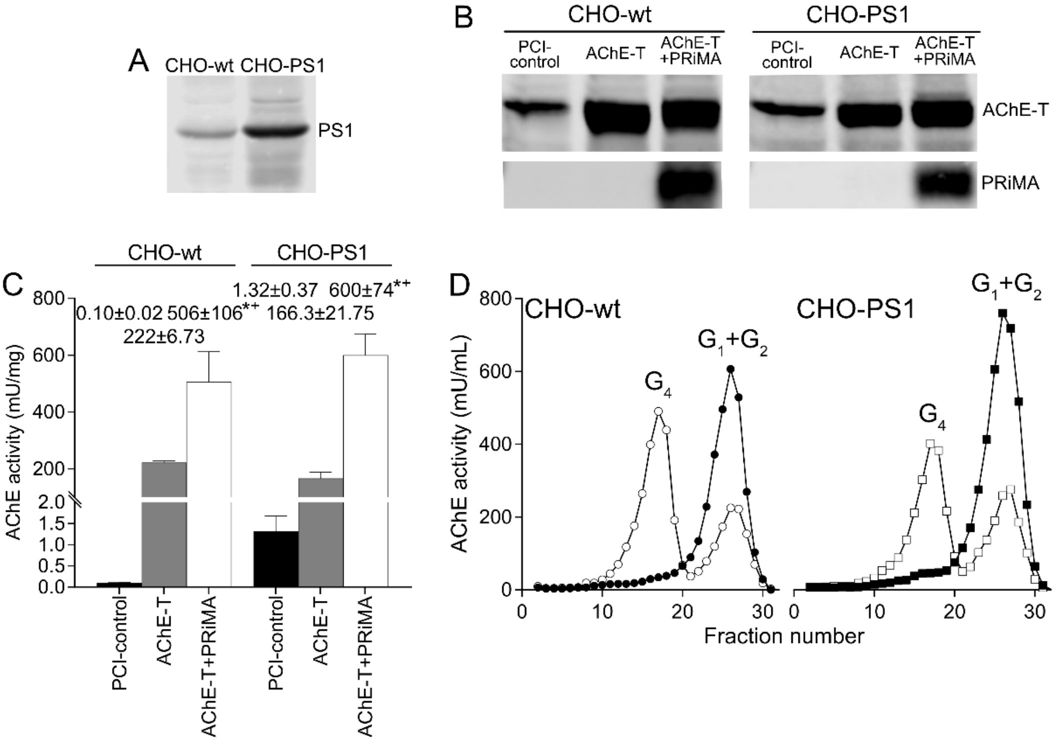

Supplemental Figure S2. Overexpression of cholinergic acetylcholinesterase "*tailed*" variant (AChE-T) in wild type Chinese hamster ovary cells (CHO-wt) and PS1 overexpresssing cells (CHO-PS1). Cells were transfected with plasmid cDNA that encodes AChE-T variant or alternatively with the AChE-T cDNA together with a plasmid cDNA encoding PRiMA1 (AChE-T+PRiMA), the anchor subunit of AChE to the membrane. As control a PCI-empty vector was overexpressed. After 48 hours cells were solubilized and AChE activity and protein assayed. (**A**) Western blot of PS1 that demonstrated the increase in PS1 levels in CHO-PS1 cells. (**B**) AChE and PRiMA1 overexpression were analysed by western blot. Representative western blots of AChE-T and PRiMA to confirm the increase in AChE 70-kDa immunoreactive band in cells transfected with AChE-T. The PRiMA immunoreactivity at 22-kDa confirms the presence of PRiMA 1 in AChE-T+PRiMA co-transfected cells. (**C**) AChE enzymatic activity was measured in cellular extracts. Graph represents mean ± SEM. * Indicates p value <0.05 respect to PCI-control transfected cells and + p value <0.05 between AChE-T and AChE-T +PRiMA when applying One-way ANOVA with Tukey's multiple comparisons. (**D**) Molecular forms of AChE in CHO-wt and CHO-PS1cells were analysed by ultracentrifugation in sucrose gradient. AChE activity was measured in collected fractions. Representative profiles of CHO-wt (circles) and CHO-PS1(squares) overexpressing AChE (closed symbols) or AChE-T +PRiMA (empty symbols) are shown. Tetrameric forms (G4) and lighter monomers and dimers (G1+G2) were identified by comparison with the position of molecular weight markers catalase (11.4S) and alkaline phosphatase (6.1S).
