# Supplementary material for: Presenilin 1 Modulates Acetylcholinesterase Trafficking and Maturation
Source: Int J Mol Sci. 2023 Jan 11;24(2):1437. doi: 10.3390/ijms24021437 (PMC9864477; doi:10.3390/ijms24021437)
Supplement: Supplementary file 1 [file ijms-24-01437-s001.zip › Supplemental Fig v2/Supplemental Figure S3.pdf]

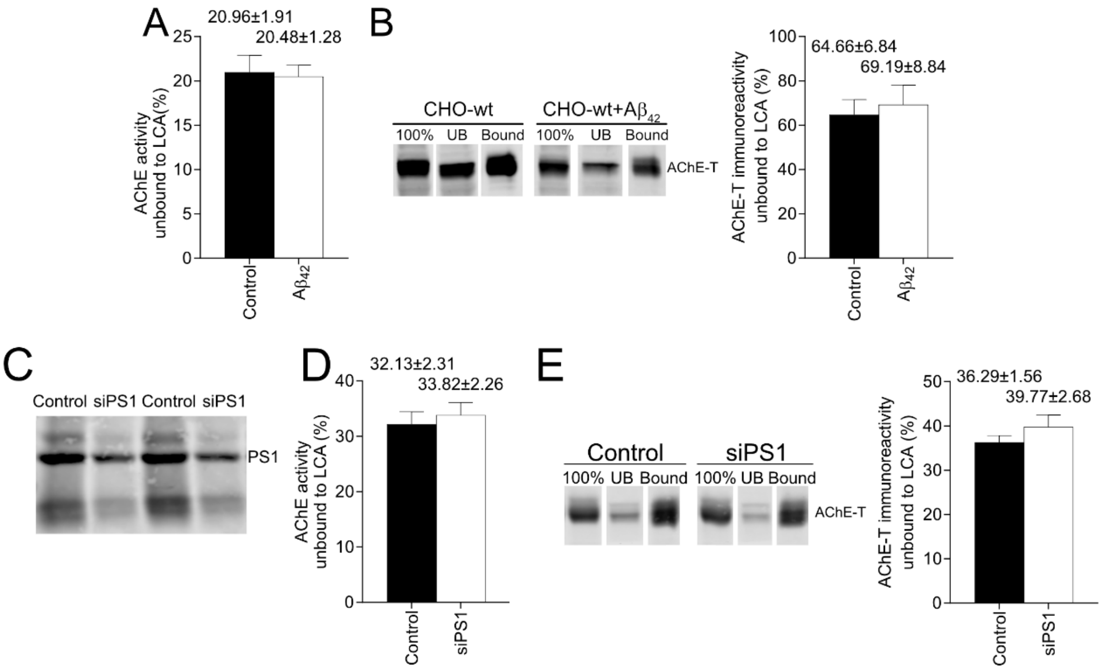

Supplemental Figure S3. Interaction of AChE of Chinesse hamster ovary cells with *Lens culinaris* (LCA) lectin. Wild type CHO cells were treated with amyloid-β 42 (CHO-wt+Aβ_{42}) and after 48 hours cells were solubilized and interaction with LCA assayed. (**A**) AChE enzymatic activity in CHO-wt+Aβ_{42} and untreated CHO-wt cells was measured in the unbound fraction to the lectin and expressed as percentage respect to enzymatic activity prior to lectin interaction. (**B**) Interaction of AChE protein was analysed by western blot of the bound and not recognized unbound fraction (UB) in comparison with total AChE before the binding (100%). Representative western blot is shown. At right, densitometric quantification of the AChE immunoreactive band not recognized by the lectin expressed as percentage respect to 100%. (**C**) In CHO-PS1 cells, PS1 expression was reduced by transfection of a PS1 siRNA for 48 hours. Representative western blot that demonstrated the reduction in PS1 in siRNA transfected cells (siPS1). (**D**) LCA interaction was done and AChE activity measured in the unbound fraction. Percentage of AChE respect to 100% was calculated. (**E**) Western blot of AChE in bound and unbound (UB) fraction was done to analyse the binding of AChE protein. Representative western blot and relative quantification of AChE immunoreactivity in the UB fraction (percentage respect to 100%) are shown. Graphs represent mean ± SEM. n=6 samples from each cell type of two independent experiments.
